# Aspects of Antiviral Strategies Based on Different Phototherapy Approaches: Hit by the Light

**DOI:** 10.3390/ph15070858

**Published:** 2022-07-13

**Authors:** Hannah Kunstek, Fanny Vreken, Aminata Keita, Michael R. Hamblin, Florence Dumarçay, Mihayl Varbanov

**Affiliations:** 1 L2CM, Université de Lorraine, Centre National de la Recherche Scientifique (CNRS), 54000 Nancy, France; h.kunstek@student.tugraz.at (H.K.); fanny.vreken6@etu.univ-lorraine.fr (F.V.); aminata.keita@etu.univ-tours.fr (A.K.); florence.dumarcay@univ-lorraine.fr (F.D.); 2 Graz University of Technology, 8010 Graz, Austria; 3 Faculté de Pharmacie, Université de Tours, 37000 Tours, France; 4 Laser Research Centre, University of Johannesburg, Doornfontein 2028, South Africa; hamblin.lab@gmail.com; 5 Laboratoire de Virologie, Centres Hospitaliers Régionaux Universitaires (CHRU) de Nancy Brabois, 54500 Vandœuvre-lès-Nancy, France

**Keywords:** phototherapy, antiviral, PDT, photothermal, photoacoustic, virus

## Abstract

The severe acute respiratory syndrome coronavirus 2 (SARS-CoV-2) which caused the COVID-19 pandemic spreading around the world from late 2019, served as a ruthless reminder of the threat viruses pose to global public health. The synthesis of new antiviral drugs, as well as repurposing existing products, is a long-term ongoing process which has challenged the scientific community. One solution could be an effective, accessible, and rapidly available antiviral treatment based on phototherapy (PT). PT has been used to treat several diseases, and relies on the absorption of light by endogenous molecules or exogenous photosensitizers (PS). PT has often been used in cancer treatment and prophylaxis, and as a complement to established chemotherapy and immunotherapy in combined therapeutic strategy. Besides significant applications in anticancer treatment, studies have demonstrated the beneficial impact of PT on respiratory, systemic, emerging, and oncogenic viral infections. The aim of this review was to highlight the potential of PT to combat viral infections by summarizing current progress in photodynamic, photothermal, and photoacoustic approaches. Attention is drawn to the virucidal effect of PT on systemic viruses such as the human immunodeficiency virus and human herpes viruses, including the causative agent of Kaposi sarcoma, human herpes virus (HHV8). PT has good potential for disinfection in anti-norovirus research and against pandemic viruses like SARS-CoV-2.

## 1. Introduction

**Figure 1:**
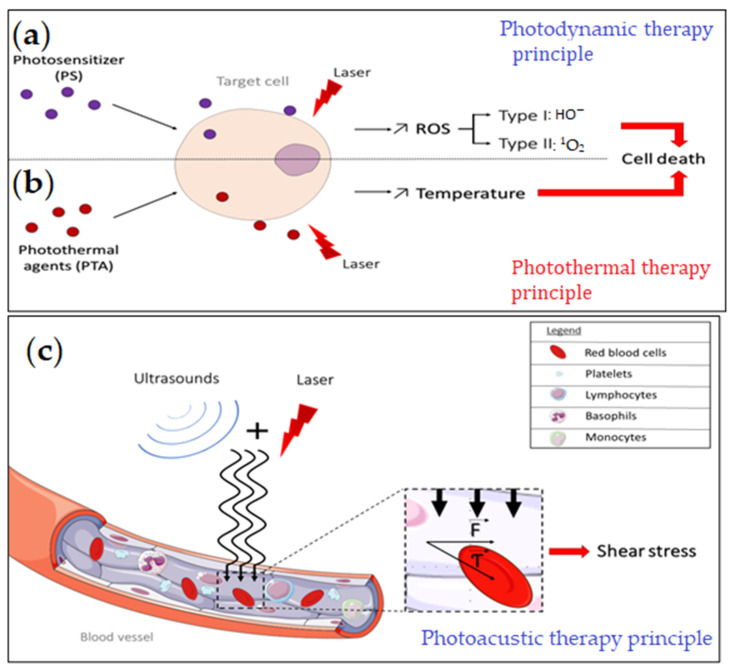
Photodynamic, photothermal, and photoacoustic therapy principle. (**a**). PDT: Excitation of the photosensitizers (PS) by different light sources (NIR laser, blue light, or incandescent light) produces reactive oxygen species (ROS) via two types of processes depending on the PS, the hydroxyl radicals (HO−) via type I and singlet oxygen radicals (1O2) via type II. The singlet oxygen (1O2) is the main cytotoxic agent involved in PDT. (**b**). PTT: Thermal phototherapy relies on the ability of organic or inorganic photothermal agents (PTA) to enter an excited state when subjected to a laser in the near infrared (NIR) wavelengths. The excited PTA will release vibrational energy which leads to a rise in temperature. (**c**). PAT: The synchronization of laser pulses and ultrasound bursts could cause a shear stress (Ƭ) in the microvessels. The shear stress is a mechanical force resulting from tangential force (F) acting on a surface, and it can directly affect the physiological function of cells in targeted tissue.

The treatment and prevention of viral infections have been the focus of scientific research, particularly since the coronavirus disease 2019 (COVID-19) pandemic [1], ([2] p. 19), [3]. It was acknowledged that viruses pose a great threat to human health, and that antiviral treatments play a crucial role in overcoming it. Some viruses can cause serious complications [4,5,6] and many undergo spontaneous mutations, which helps them render vaccines and treatments ineffective [7,8]. Therefore, it is essential to develop new and innovative antiviral therapies. Phototherapy has proven its worth both in medical imaging and anticancer treatment [9,10,11]. Photodynamic (PDT) and photothermal (PTT) therapies require the use of photosensitizers (PS) and photothermal agents (PTA), respectively. PS and PTA are molecules that can be photoactivated and if delivered to the organism, can cause targeted cellular damage. The major difference between PTT and PDT, is that PTT causes a temperature rise which can increase the tissue and cell damage (Figure 1a) [11,12]. In PDT, there are reactive oxygen species produced, which when accumulated, are detrimental to the target cell (Figure 1). Another approach we discuss is photoacoustic therapy (PAT), which allows both imaging and treatment at the same time. In this approach, both laser and ultrasound contribute to the shear stress in target tissue and/or cells (Figure 1b) [13]. 

**Figure 2:**
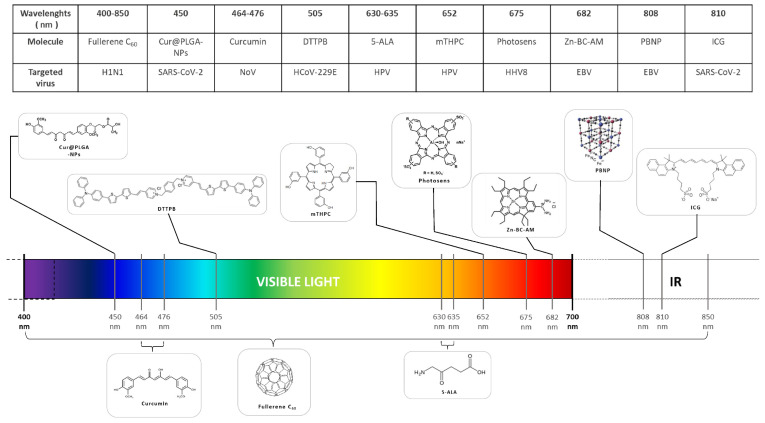
Photosensitizing molecules used in antiviral phototherapy. Organic or nonorganic molecules irradiated in visible and/or infrared wavelengths displayed antiviral activity. Assigned to each molecule are the irradiation wavelength and a target virus.

These approaches might allow direct in vivo targeting of infections caused by viruses. So far, there have been some promising data gathered regarding phototherapy applications in antiviral treatment (Table 1) (Figure 2). Particular effectiveness was noted for enveloped viruses, as it has been shown they tend to be more sensitive to light-inactivation (Table 1) (Figure 2). 

### 1.1. Photodynamic Therapy (PDT)

Discovered over 100 years ago, photodynamic therapy (PDT) is a noninvasive method based on photochemistry. PDT involves the use of photosensitizing agents, or dyes, coupled with light with an appropriate wavelength to be absorbed, and surrounding oxygen. (Figure 1a). The reaction leads to the excitation of the photosensitizer and the production of reactive oxygen species (ROS), in particular, excited state singlet oxygen (^1^O_2_). In the context of tumor treatment, PDT-produced ROS have been shown to be effective in numerous clinical trials. Recent studies have shown that the ROS could also target the viral envelope [14,15] and could therefore be used as an antiviral treatment. The efficiency of the photosensitizer depends on the height of the absorption peak, and the yield of the excited triplet state. Near infrared light (NIR) or far-red light with a wavelength between 620 nm and 850 nm is able to penetrate deeper into tissue, which makes it the best phototherapeutic window. Effective light sources include lasers, light-emitting diodes (LEDs), and filtered incandescent light [15,16]. A review by Wiech et al., 2019, provides extensive information on the activity of different classes of photosensitizer on various viruses [17]. Most of the dangerous, persistent, or emerging viruses are enveloped viruses [16]. Studies have shown that these viruses are particularly sensitive to PDT because their lipid envelope is easily oxidized [16].

### 1.2. Photothermal Therapy (PTT)

Photothermal therapy (PTT) is a minimally invasive technique, which causes a targeted increase in temperature with the goal of killing abnormal or unwanted cells in the vicinity. PTT is mostly used as an anticancer treatment, but recently, antiviral PTT has been explored. The PTT principle consists of injecting or applying nanoparticles or other highly absorbing materials locally into the target tissue and then irradiating with a laser, often with a short pulse duration (Figure 1a). Thanks to their high absorption coefficients, photothermal agents (PTA) are activated by the irradiation, which sharply raises their vibrational energy. This locally increases the temperature of the tissue to be treated, inducing cell death when the temperature exceeds 60 °C [12]. Current research has focused on new inorganic or organic photosensitizing agents with biodegradable properties and low toxicity [18]. Longer wavelength light is less energetic and thus less harmful for neighboring cells. Studies have shown that use of a second near-infrared window (NIR-II) 1000–1700 nm allows deeper tissue penetration and more efficient and accurate PTT with less damage to other cells and tissue [19,20].

### 1.3. Photoacoustic Therapy (PAT)

Ultrasound-based therapies, such as antivascular ultrasound therapy (AVUT) and shock wave-enhanced emission photoacoustic streaming (SWEEPS) have been investigated for destruction of abnormal microvessels and in dental procedures, such as root canal cleaning. Similar to SWEEPS, photo-mediated ultrasound therapy (PUT) uses both laser pulses and ultrasound bursts. It is a noninvasive and agent-free approach, which relies on a photoacoustic cavitation mechanism to close microvessels [20]. The principle is believed to rely on the synchronization between the laser pulses and the ultrasound blast, which could cause an increased shear stress in microvessels and directly affect the physiological function of cells in the targeted tissue [13] (Figure 1b). PUT is highly localized and precise, and allows the targeting of affected tissue, and to tailor the depth of the treatment [13]. The illumination of the targeted tissue results in local thermal heating and elastic expansion, thus generating photoacoustic or PA waves. The generated PA waves may not only be useful for therapy, but can also be detected using ultra-high frequency transducers outside the body [21].The detected signals can be processed to obtain high resolution PA and ultrasound images [13,22,23]. PA imaging (PAI) allows higher spatial resolution and deeper tissue penetration than other optical imaging approaches [22,23]. Furthermore, contrast enhancement can be achieved by the addition of imaging agents [22]. There are four mechanisms of action that could be involved either singly or combined: cavitation, radiation pressure, acoustic streaming, and ultrasound-induced heating [24]. In the cavitation mechanism, small bubbles are formed under the ultrasound pressure. A laser-generated focused ultrasound (LGFU) system with high frequency, tight focal spot, small aperture, and reduced ultrasound-induced heating was successfully used in anticancer drug delivery [24]. Besides anticancer treatment applications [24], LGFU and PAT could be used for the treatment of different viral infections by directly targeting the infected site.

## 2. Respiratory Viruses

Human respiratory viruses infect the human respiratory tract and may have either an RNA or a DNA genome. These viruses can be either enveloped or nonenveloped virions [25]. Symptoms can range from a common cold to fatal pneumonia. Elderly or immunocompromised individuals, as well as children and infants, are most vulnerable to severe infection. Transmission is mostly by human-to-human contact through inhalation of infectious droplets [26]. Following the global epidemic of COVID-19, antiviral treatments have been the focus of much scientific research. Despite the development of vaccines, some viruses like influenza continue to undergo recurrent antigenic variations [27] which is why photodynamic therapy may have a major advantage in the future. 

A study by Majiya et al. demonstrated that nonenveloped viruses are less sensitive to photodynamic inactivation (PDI) than enveloped viruses. Using the photosensitizer tetrakis (1-methyl-4-pyridinio) porphyrin-tetra-p-toluene sulfonate (TMPyP) on different nonenveloped viruses as a viral photo-inactivation technique, they found that the inactivation of the viruses depended on the abundance and accessibility of singlet oxygen sensitive amino acids in the envelope [28]. Studies have shown the ineffectiveness of certain photosensitizers such as naphthalene or orthoquine against nonenveloped viruses such as adenoviruses [17,29] and the photosensitizer meso-tetraphenylporphine with two sulfonate groups on adjacent phenyl rings (TPPS_2a_) could even increase the presence of the virus in the cell nucleus [30]. 

For enveloped viruses, photodynamic inactivation has been proven using photosensitizers that produce reactive oxygen species to damage the viral envelope and degrade the lipids, thus lessening the infectivity of the virus [16]. Pourhajibagher et al. [31] reported the use of curcumin-loaded polymeric nanoparticles and blue light to sterilize plasma products. They were able to show the ability of their nanoparticles to inactivate the SARS-CoV-2 virus in contaminated patient plasma by formation of ROS, and the non-cytotoxic effect of the treatment and laser on Vero cells in vitro. Other disinfection studies on plasma or blood bags using PDI showed the inactivation of different viruses, such as coronavirus or influenza virus with photosensitizers such as curcumin [31], methylene blue, crystalline fullerene C_60_ [32], NT-P (porphyrin-conjugated multiwalled carbon nanotubes) [33]. 

Wu et al. [34] developed a model mimicking the cell membrane, using photosensitizers with aggregation-induced emission characteristics (DTTPBs) that mimic the phospholipid structure in order to prevent the attachment of cells to the membrane and thus allow the elimination of viruses by ROS formation upon irradiation with white light. In tests carried out on the HCoV-229E virus using this method, they found that viral RNA or HCoV-229E proteins were not found within the cells, thus showing the effectiveness of the treatment. 

There are also other photosensitizers, which can affect the attachment of the virus to the cells, such as indocyanine green (ICG). In the case of SARS-CoV-2-RBD, studies have been conducted by Pourhajibagher et al. on the inactivation of this virus using ICG. RBD is a protein with a receptor binding domain and therefore plays a role in the entry of the virus into cells via the surface spike proteins interacting with the cell receptor ACE2 (angiotensin-converting enzyme 2). This study demonstrated an interaction between the RBD of SARS-CoV-2 (SARS-CoV-2-RBD) and ICG, allowing inactivation of the virus, but the studies lacked any in vitro or in vivo data [35]. Photodynamic inactivation of parainfluenza-3 virus (hPIV3) was performed with phthalocyanines (Pcs) as photosensitizers [36]. The irradiation wavelength in the far-red light spectrum produced different ROS that allowed viral inactivation. 

Other viruses can affect the respiratory tract, but in these cases, it is the symptoms that are treated by PDT. Infection with human papillomavirus (HPV) types 6 and 11 can lead to RRP (recurrent respiratory papillomatosis), which is a chronic disease leading to the formation of benign epithelial tumors in adults and children. These tumors are located in the aerodigestive tract and can become malignant [37]. Generally, patients with tumors due to HPV infection undergo surgery to remove the tumors, but recurrence is almost inevitable. In the literature, it has been shown that these epithelial tumors can be treated with photodynamic therapy and trials have been carried out on children with RRP. Photodynamic therapy was performed during three separate visits on the subjects with direct application of the photosensitizer, aminolevulinic acid hydrochloride with irradiation at 100 to 200 J/cm^2^ at 635 nm for 30 min at the lesion. Patients were free of recurrence 6 months after treatment [38]. Another study treated a patient who had undergone multiple surgical removals with recurrence, with a combination of CO_2_ laser removal of the lesions plus PDT using ALA applied to the surgical site followed by 30 min of 635 nm irradiation at a dose of 120 J/cm^2^. There was no recurrence at 15 months post-treatment [39]. 

The applications of PT using excited PSs for treating respiratory virus infections by targeting viral envelope are listed in Table 1.

## 3. Systemic Viruses and Sexually Transmitted Infections

An infection is said to be systemic when it spreads to several systems of the body. Many sexually transmitted infections (STIs) are systemic infections or can become systemic infections over time.

The human immunodeficiency virus (HIV) is the causative agent of acquired immunodeficiency syndrome (AIDS) and is a sexually transmitted infection that attacks the immune system. HIV is a virus in the family *Retroviridae* of the genus lentivirus. It includes two types: HIV-1 and HIV-2. Its genome is composed of two single-stranded RNA sequences whose organization differs between the two types [40]. The current treatment of HIV is based on triple therapy consisting of two nucleoside reverse transcriptase inhibitors combined with either a protease inhibitor, an integrase inhibitor, or a nonnucleoside reverse transcriptase inhibitor, which actively suppresses virus replication in the body [41]. This triple therapy involves lifelong adherence to an anti-retroviral regimen. Nonadherence to the regimen alters the efficacy of the drugs and increases drug resistance, and this poses a threat to virus spread, not to mention toxicity, adverse effects, and the establishment of viral reservoirs [26,42]. Several long-acting formulations are in clinical development, but the combination of cabotegravir and long-acting injectable rilpivirine, an investigational integrase inhibitor and a non-nucleoside reverse transcriptase inhibitor, respectively, was approved in Canada in 2020 [43]. A study by Huijuan et al. investigated the inhibitory effect of hematoporphyrin monomethyl ether-mediated PDT (HMME-PDT) on cell-free HIV viral particles with promising results [44]. HMME is a photosensitizer with low toxicity, having a good photodynamic effect as it produces enough singlet oxygen with fewer side effects. The results showed that HMME-PDT could inactivate up to 100% of HIV-1 and HIV-2 viral particles. Resistant strains of HIV were fully susceptible to PDT [44]. 

HIV infection is often associated with certain opportunistic infections, either bacterial, fungal, or viral; in particular, certain viruses of the *Herpesviridae* family. The *Herpesviridae* family is composed of viruses infecting humans (HHV) and others infecting animals [45,46]. One of the properties common to all herpes viruses is their ability to remain in a latent form in host cells, but then reactivate and replicate by cell lysis [45]. 

Kaposi’s sarcoma (KS) is a multifocal angioproliferative neoplasm caused by HHV8 infection that often occurs in immunodeficient individuals. Frequently, HIV-infected individuals are co-infected with HHV8, and the development of KS can lead to mortality in AIDS patients. Pegylated liposomal doxorubicin, liposomal daunorubicin, and taxane paclitaxel are Food and Drug Administration (FDA)-approved therapeutic drugs, but their efficacy decreases over time [47]. Bernstein et al. used PDT with photofrin (630 nm) on 25 patients living with HIV who developed KS. The results were considered positive without any alteration of the patient’s immune system [48]. In the case of classical KS, i.e., without HIV, the efficacy of PDT using Photosens as a photosensitizer (675 nm) was demonstrated in a 79-year-old patient with tumors in the ankle area. A decrease in tumor thickness and lesion area, and emergence of smooth skin 4 months after PDT were noted [49].

Herpes simplex viruses (HSV-1 and HSV-2) are transmitted by various routes and also have different targets within the body. Primary infection with HSV-1 often goes undetected because the virus remains in a latent state. Reactivation of HSV-1 results in herpes labialis, neonatal ocular and central nervous system herpes, while HSV-2 infection causes genital herpes [50]. Acyclovir, foscarnet, and penciclovir are direct-acting antiviral drugs used for the treatment of HSV infections. In an attempt to overcome viral drug resistance, Andrea et al. conducted a study on orthoquin, a plant extract acting as a photosensitizer with antiviral activity. Orthoquin-mediated photodynamic inactivation inhibited HSV-1 and HSV-2 infection of cells in a dose-dependent manner [29]. An in vitro study by Zverev and colleagues demonstrated the antiviral efficacy of PDT with the photosensitizer Fotoditazine^®^ (10 µg/mL) in the treatment of herpes infections (HSV-1 and HSV-2) in Vero cells, as the viral titer was decreased more than two orders of magnitude upon irradiation (1.8 J/cm^2^, 30 s) [51].

Cytomegalovirus (CMV) is a member of the herpesvirus family (HHV5) that can cause fatal diseases [46]. A PTT-based antiviral strategy against CMV infection was based on the conjugation of a monoclonal antibody directed against the surface glycoprotein of CMV to gold nanoparticles. Following exposure to a 670 nm laser at 1.5 W/cm^2^, the gold nanoparticles generated heat up to 50 °C which selectively killed the infected cells [52].

Epstein–Barr Virus is a virus of the *Herpesviridae* family (HHV4), which has been associated with several malignancies, including Burkitt’s lymphoma. A study was conducted by Burga and colleagues using nanoimmunotherapy based on PTT. This study consisted of conjugating Prussian blue nanoparticles (PBNP) to Epstein–Barr virus (EBV) antigen-specific cytotoxic T lymphocytes (CTL) as a PTT agent, that were then irradiated with a NIR laser. They obtained promising results because PBNP-coated CTLs efficiently lysed target cells expressing EBV antigen, compared to uncoated CTLs [53]. 

Several studies have been conducted on the use of PT in the treatment of lesions or cancers caused by human papillomavirus. HPV infection is associated with several cancers, including cervical cancer, condyloma, ano-genital cancer, oropharyngeal cancer, and some warts. HPV is a double-stranded DNA virus, which codes for some human oncoproteins that account for its oncogenic characteristics [54]. Treatment of the infection is based on the type of lesion or cancer [55]. Lesions can be treated by laser ablation with the carbon dioxide laser with a wavelength of 10,600 nm, or by 5-aminolevulinic acid (topical ALA) PDT using 630 nm or 635 nm light [56,57]. The efficacy of PDT in the treatment of HPV-related diseases has been described in several reviews [56,57,58]. 

Hepatitis B virus (HBV) is a partially double-stranded DNA virus infecting hepatocytes. Chronic HBV infection is associated with liver cirrhosis and hepatocellular carcinoma. Current treatment is based on interferon, adefovir, and lamivudine, which have risks of developing long-term resistance. Ailioaie et al. reported that PDT with a blue laser (400–470 nm) and curcumin (derived from turmeric spice) could be an alternative approach in chronic HBV and hepatocellular carcinoma. This is because prolonged exposure to blue light inactivates viruses and curcumin affects various cell signaling pathways by amplifying the ability of the immune system to eliminate tumor cells [59].

## 4. Central Nervous System Viral Infections

Acute central nervous system (CNS) viral infections are a cause of high morbidity and mortality [60]. CNS infections are mostly caused by viruses from *Herpesviridae* and *Picornaviridae* families, as well as arboviruses, and less common viruses such as mumps caused by two members of *Paramyxoviridae*, *Orthorubulavirus*, or *Morbillivirus *[61]. Additionally, zoonotic viruses, such as rabies and lymphocytic choriomeningitis virus [61,62], or emerging viruses, such as Ebola and West Nile virus, can infect the CNS and cause neurological complications [61,62]. Zika virus (*Flaviviridae*, Flavivirus) for example, has also been linked to congenital CNS malformations, such as microcephaly [61]. CNS infections primarily manifest as meningitis or encephalitis, which is accompanied by mental, motor-sensory, and behavioral changes [62,63]. Despite nonstandardized reporting policies and differences in surveillance systems, the incidence of CNS infections is reported to be increasing [61,64,65]. Current treatment consists mostly of corticosteroids [66] and antiviral drugs, such as acyclovir [66] or valganciclovir [63,67]. For some viruses, prophylaxis in the form of a vaccine is available; however, most CNS viral pathogens lack an effective, noninvasive therapy [60]. Besides the difficulty of obtaining a correct diagnosis [61,66], the major challenge of an effective therapy is the difficulty of drug delivery to CNS due to the blood-brain barrier, which can be disrupted by multiple factors [60,68].

So far, there have been only a few attempts to study PDT/PTT and/or PAT in CNS viral infections. Nevertheless, there is some promising data that might point towards further specific research. Menon et al. reported a reversal of brain dysfunction following 24 weeks of UV-A1 irradiation (340–400 nm filtered wavelength, 160 kJ/ m^2^, for 30 min) [69]. PDT with Zn (II)-benzochlorin analog (Zn-BC-AM) had a significant effect (*p* < 0.05) on proinflammatory cytokine production (3.5–4-fold increase of IL-1α; 5-fold increase of IL-1β, and approximately 80% decrease of IL-8) in HK-1-EBV cells, which suggests the efficacy of PDT in the treatment of EBV-associated nasopharyngeal carcinoma [70]. In a study by Wang et al., the selective inhibition of inflammation in the CNS in an experimental autoimmune encephalomyelitis (EAE) mouse model was reported after UVB (300–315 nm) irradiation [71]. Additionally, there was a decrease in demyelination and inhibition of chemokine CCL5 production in the spinal cord [71]. Because some viral infections lead to demyelination, PDT could be a promising solution. Furthermore, a technique called transcranial photo-biomodulation with near-IR light displayed promising preliminary results in improving anxiety disorder symptoms [72], and could potentially be considered to improve behavioral changes caused by CNS viral infections. Low-level light therapy (LLLT) or photo-biomodulation has also been successfully tested as a safe, noninvasive and inexpensive alternative for modulating neurological and physiological processes, as well as attenuation of neuroinflammation and prevention of ischemic brain injury in mice [73]. A study by Semyachkina-Glushkovskaya et al. showed promising results of PDT (635 nm laser at a dose of 15 J/cm^2^ plus 20 mg/kg 5-aminolevulinic acid) to temporarily increase blood-brain barrier permeability in mice [60]. This method could allow more effective delivery of drugs to the CNS, and therefore might improve treatment of the disease.

## 5. Gastroenteric Infections

There are more than 685 million cases of acute viral gastroenteritis reported annually [74]. Viral gastroenteritis (GE), also called “stomach flu”, results in up to 200,000 deaths of children each year, which is one of the highest pediatric mortality rates worldwide [75,76]. Enteric adenoviruses (eAdVs), rotaviruses (RV), astroviruses (HAtVs), and sapoviruses (SaV) are a leading cause of acute GE, along with noroviruses (NoV) [74,77,78]. These viruses do not belong to the same family, but are all nonenveloped, with a protein capsid and icosahedral geometry [77]. Additionally, they are transmitted fecal-orally, and through contaminated water or food [77]. The infection usually clears within a few days, and thus, in most cases, is not life threatening. Therefore, not many specific treatments are available. However, due to potential complications [79], and high mortality in children under 5 years of age, some oral RV vaccines have been developed for infants. It has been shown that despite an increase in viral genetic diversity, the vaccine efficacy remained unaltered [80]. On the other hand, it has been pointed out that vaccines may have a higher risk for immunocompromised [81,82] or malnourished [83] children. In addition, there have been some technical limitations that have had a negative impact on accessibility of vaccines in certain parts of the world [84]. The GE therapy research is limited, as there are some difficulties with growing certain viruses and/or specific strains [85]. There are not so many new antivirals and treatments found, as more attention has been on repurposing drugs [86], such as nitazoxanide [87,88,89,90,91], rupintrivir, and citrate among others [76,85,92,93,94]. However, none of the potential treatments has been introduced as accepted therapy [76].

Because there are some challenges and limitations of current GE therapies, the search for appropriate, effective, and widely accessible therapy continues. A study by Wu et al. showed that photoactivated curcumin, a polyphenolic extract and a common food additive, could alter the morphology and inactivate NoV contamination in oysters [95]. Randazzo et al. assessed the activity of photoactivated curcumin against two human NoV surrogates, feline calicivirus (FCV-F9) and murine norovirus (mNoV) [96]. The antiviral effect was evaluated under different conditions, and it was observed that 30-min exposure to blue LED light led to complete inactivation of diluted FCV-F9 treated with 50 μg/mL curcumin. There was a correlation between virus titer and curcumin efficacy. While curcumin treatment had a significant effect (*p* < 0.05; 1.75 log reduction at room temperature, and 4.43 log reduction at 37 °C) on FCV titers, it showed only a slight effect on mNoV. Nevertheless, photo-inactivation could be effectively used in combination with other antiviral approaches, such as sonication. A study by Su et al. tested the efficacy of high-intensity ultrasound (HIUS) on three standard human NoV surrogates, including FCV, mNoV, and MS2 bacteriophage [97]. The study showed a correlation between HIUS inactivation and viral titer. Additionally, the degree of HIUS inactivation was dependent on the virus type and exposure time. At lower titer, after 10 min sonication, there was an inactivation of FCV and MS2, while mNoV at lower titer was successfully inactivated after 30 min. For high titer viruses, longer sonication was needed to achieve the same reduction levels for both FCV and MS2. When the viruses were diluted in orange juice instead of phosphate buffered saline (PBS), there was a lower efficacy of HIUS inactivation. Despite not meeting the FDA standard of 5 log reduction in orange juice, the method generally showed promising results and provided clear evidence of positive effects.

## 6. Eye Infections

Besides the respiratory tract, mouth, and genitalia, eyes are the most common site of viral infections [98]. It is estimated that out of approximately 1.5 million patients suffering from a viral eye infection, each year 40,000 end up with severe eye damage [99,100]. The most prominent causes of eye infections are the systemic herpes simplex (HSV-1) and varicella zoster viruses (VZV) [98,99,100], that tend to lie dormant within the sensory spinal or trigeminal ganglia [98,101]. Similarly, infections can be caused by rubella (RuV) or cytomegalovirus [102], among others [103]. To reduce the recurrence and possible complications [101,104], prompt therapy is needed as soon as the symptoms appear [102]. Antiviral drugs are commonly prescribed (acyclovir or valacyclovir) alongside topical corticosteroids [98] and/or cycloplegic/mydriatic drops for pain and inflammation. A trabeculectomy may be needed in cases of a severe increase of intraocular pressure (IOP) in glaucoma patients [102]. With infection being mostly lifelong, patients suffering from chronic eye infections are often dependent on continuous treatment. However, long-term corticosteroid therapy could have a significant effect on the general health of patients [105]. On the other hand, lasers have been used for eye treatments and vision correction for many years. Retinal photocoagulation [106] is a rapid and precise laser method that causes small burns around the retina and seals the retina to the eye wall, to prevent it from further detachment. Despite the efficiency of the treatment, the scarring caused by photocoagulation is permanent [107]. Similar to the photo-coaglutination method, a PDT method using 6 mg verteporfin [108,109] plus 690 nm light has been shown to seal the blood vessels and prevent further retinal damage. The major advance of the PDT approach is the precision of the laser with no heat production, which minimizes the damage to surrounding tissue. On the other hand, a 2005 case report by Klais et al. pointed towards the risk of acute vision loss or eyesight worsening upon PDT treatment of choroidal neovascularization (CNV) [109]. Paulus et al. obtained promising data by testing the effect of photo-mediated ultrasound therapy in a CNV rabbit model [107]. CNV-induced rabbits were treated with 10 Hz laser pulses (14% at 532 nm and 86% at 1064 nm) with ultrasound bursts of 0.5 MPa intensity. At 24 h post treatment, there were no visible morphological changes in the retina, but blood clots within the choroidal vasculature lumen were observed in some cases. The treatment proved to be selectively effective against neovascularization without damage to surrounding tissue [109]. The data suggested this was a safe and potentially effective therapy for neovascularization, which has also been linked with HSV-1 infection [110]. Therefore, PUT might be considered useful in combination with antiviral drugs to fight eye infections. (Figure 2).

## 7. Skin Infections

Viral skin infections affect the mouth and limbs locally, but they can also be systemic. Most common viruses that cause skin conditions include *Poxiviridae*, *Paramyxoviridae*, *Papilomaviridae* and *Herpesviridae* families. Symptoms [111] can vary from rough bumps on the skin [112,113,114] to pain, numbness, or a tingling sensation in the affected area [115]. Viral skin infections are more common in children and immunocompromised patients, and one of the most prevalent ones are HPV-caused verrucae. Verrucae or warts tend to be treated with cryotherapy or salicylic acid [111]. Cryo-techniques include liquid nitrogen, dimethyl ether, or propane, that cause cell damage and increase a local inflammatory response [116]. Dimethyl ether and propane are more accessible, but their efficacy is significantly lower than the one from liquid nitrogen [113]. Generally, cryotherapy is not recommended for younger patients, as it has been commonly associated with pain and discomfort [113,114]. Warts can also be treated with salicylic acid [117], a natural plant extract with anti-inflammatory and antibacterial properties [113]. Despite its good efficacy, salicylic acid treatment requires daily application over the course of a few weeks and/or months. Moreover, it is not recommended to patients suffering from nerve damage or arterial narrowing [113]. Some other, less effective topical methods include treatment with formaldehyde, glutaraldehyde, and silver nitrate among others [111,114,116]. All mentioned therapies are widely used, but neither is suitable for treating resistant plantar warts [113,117]. Warts, alongside other skin infections, can have a severe impact on health and viral transmission [113,114]. Despite this, most prescribed therapies are topical, retroactive treatments of skin lesions [111,113,116], with only few therapies focusing on HPV and viral particle transmission [113]. Therefore, there is a need for novel cause-oriented, accessible, effective, and safe therapy for both younger and older patients. 

**Table 1:** Different phototherapy approaches used to treat viral infections.

Respiratory Infections		Systemic & Sexually Transmitted Infections	
Viral disease and causative virus	Phototherapy approach	Viral disease and causative virus	Phototherapy approach
Coronavirus (HCoV) Common cold, Pneumonia	Blue light photoactivated curcumin-poly nanoparticles to treat plasma products contaminated by SARS-CoV-2 [30] White light irradiation photoactivated DTTPB to inactivate HCoV-OC43 and HCoV-229E in MRC-5 cells [34] Light irradiation NIR at a wavelength of 810 nm photoactivated ICG induces a decrease of SARS-CoV-2 virus attachment to the host cell surface [35]	Epstein–Barr Virus (EBV) EBV-associated cancer	NIR laser photoactivated CTL: PBNP agent, which can target and lyse EBV antigen expressing cells is a promising anticancer immuno-phototherapy approach [53].
Human papillomavirus (HPV) Recurrent respiratory papillomatosis (RRP), HPV-induced tumors	Photoactivated dihematoporphyrin ether (DHE) or aminolevulinic acid hydrochloride (5-ALA) can significantly decrease RRP growth in patients. PDT approach with m-tetra(hydroxyphenyl) chlorine is reported to be effective against HPV-induced tumors causing minimal tissue damage and less photosensitivity in rabbits. HPV-induced epithelial tumors can be treated with photodynamic therapy with the ALA molecule [37,38,39].	Human papillomavirus (HPV) Condyloma acuminata	Treatment with topical aminolevulinic acid photodynamic therapy (ALA-PDT) following curettage was effectively applied for the treatment of condyloma acuminate [56,58]
Influenza Respiratory infection	Visual light (400–850 nm) photoactivated crystalline fullerene C60 in allantoic fluid of chicken embryos decreased the titer of influenza A (H1N1) [32]. Visible light from compact fluorescent lamp photoactivated NT-P on NCI cells for influenza A (H3N2) inactivation [33].	Human Herpesvirus 8 (HHV8) HHV8-induced tumor	The use of Photosens^®^ (NIOPIK, Russia) as a photosensitizer, irradiated by laser at a wavelength of 675 nm, resulted in a decreased thickness of the tumor and the lesion area. After 4 months of treatment, the patient regained smooth skin [49].
CNS Infections		Skin Infections	
Viral disease and causative virus	Phototherapy approach	Viral disease and causative virus	Phototherapy approach
Epstein–Barr Virus (EBV) EBV associated nasopharyngeal carcinoma	Zn-BC-AM PDT had a significant impact on proinflammatory cytokine production in HK-1-EBV cells [70].	Human papillomavirus (HPV) Verrucae	Long-pulsed laser (1064 nm) had a verrucae vulgaris clearance rate of 96% [118]. PDT was successfully used in treatment of resistant verrucae plantaris [119]. Pulsed dye laser treatment (585 nm) had 60% clearance efficacy in children under 12 years [120]. Palmoplantar warts were cleared up to 97% upon treatment with moisturizing cream and irradiation with long-pulsed laser (1064 nm) [121]. Both conventional and daylight ALA-PDT cleared 70 to 80% facial flat warts in children [117].
Gastroenteric Infections			
Viral disease and causative virus	Phototherapy approach		
Norovirus (NoV) Gastroenteritis	UVB photoactivated curcumin showed an effect on NoV surrogates; it also altered the morphology and inactivated NoV in oysters [94,95]. There was an inactivation effect of high-intensity ultrasound on NoV surrogates noted [96].		

Phototherapy has been part of multiple dermatology studies. Irradiation with a long-pulsed laser (1064 nm) had a 96% clearance rate, which is up to 20% higher than some conventional therapies [118]. Han et al. noted complete verruca vulgaris clearance in 73% of patients after only one treatment [118]. Fadel et al. successfully deployed PDT in fighting off the resistant plantar warts [119]. Promising data was also shown in pediatrics, with a wart clearance rate above 70% [114]. Park et al. tested a pulsed dye laser (PDL) treatment (585 nm, fluence of 7.00–10.00 J/cm^2^) in children aged 12 or less [120]. The study reported 60% efficacy response and suggested the use of PDL in combination with other topical treatments, as it was found to be the safest and most tolerable among children [121]. Alshami and Mohana reported 97% palmoplantar warts clearance rate in patients (5–67 years old) upon treatment with moisturizing cream and irradiation with long-pulsed laser (1064 nm, Nd:YAG, fluence of 200–250 J/cm^2^) [121]. The clearance rate was affected by the size, position, age, and number of warts [121]. A study by Borgia et al. compared conventional to daylight ALA-PDT in treating facial flat warts in children [117]. Conventional ALA-PDT (10% ALA, 630 nm) had more rapid response, but the general therapeutic performance was superior in daylight ALA-PDT (10% ALA, 2 h daylight exposure). With a clearance rate of approximately 70 to 80%, PDT was found effective against facial flat warts regardless of the irradiation source [117]. One of the greatest benefits of PDT, is the noninvasiveness, safety, and lack of severe and/or permanent side effects and/or scaring. Additionally, as HPV has been shown to be heat sensitive [117,120], PDT and PTT might be considered a great alternative to more invasive warts treatments. 

## 8. Conclusions

Light-based therapeutic approaches are characterized by the ability to focus the beam of light onto the lesion or the part of the body that requires treatment. This has been often taken advantage of in the treatment of cancer, where the selectivity provided by the spatial confinement of illumination reduces the damage to surrounding normal tissue. However, light-based treatments can also be useful in the case of localized infections, and for sterilizing various products, such as blood products, medical devices, and foodstuffs. Every known class of pathogen can be inactivated by the appropriate PT approach, including bacteria, fungi, viruses, protozoa, parasites, and even prions. For viral infections, the reactive oxygen species produced by PT can either damage the virus structure itself and/or the host cells that are infected by the virus. Enveloped viruses which contain protein and lipids in the envelope are more easily oxidized by ROS compared to nonenveloped viruses. It is still uncertain to what extent PT approaches may be useful in systemic viral infections, but PDT has been shown to activate the host immune response in cancer treatment, and this may also apply to trigger the immune response against viruses. Antiviral phototherapy and photoacoustics, including photoacoustic molecular imaging, are two very promising approaches to fight against viral infections. Despite the potential of the photoacoustic approach, the data in the literature is still scarce. Antiviral PAT, as one of the latest phototherapy techniques, still requires more development, in order to become a precise and effective treatment. Nevertheless, photoacoustic antiviral treatment could allow the precise destruction of virus-infected cells, and minimize the damage to the surrounding normal tissue and cells.

## Data Availability

Not applicable.

## Abbreviations

^1^O_2_	singlet oxygen radicals
ACE2	angiotensin-converting enzyme 2
AIDS	acquired immunodeficiency syndrome
ALA	aminolevulinic acid hydrochloride
AVUT	antivascular ultrasound therapy
CMV	cytomegalovirus
CNS	central nervous system
CNV	choroidal neovascularization
COVID-19	coronavirus disease 2019
CTL	cytotoxic T lymphocytes
DNA	deoxyribonucleic acid
eAdVs	enteric adenoviruses
EAE	experimental autoimmune encephalomyelitis
EBV	Epstein–Barr virus
FCV	feline calicivirus
FDA	The Food and Drug Administration
GE	gastroenteritis
HAtVs	astrovirus
HBV	hepatitis B virus
HCoV	human coronavirus
HHV	human herpesvirus
HIUS	high-intensity ultrasound
HIV	human immunodeficiency virus
HMME-PDT	hematoporphyrin monomethyl ether combined with PDT
HO-	hydroxyl radicals
hPIV3	parainfluenza-3 virus
HPV	human papillomavirus
HSV	herpes simplex virus
ICG	indocyanine green
IOP	intraocular pressure
KS	Kaposi’s sarcoma
LGFU	laser-generated focused ultrasound
LLLT	low- level light therapy
mNoV	murine norovirus
NIR	near-infrared light
NoV	noroviruses
NT-P	porphyrin-conjugated multiwalled carbon nanotubes
PTA	photothermal agent
PAI	photoacoustic imaging
PAT	photoacoustic therapy
PBNP	Prussian blue nanoparticles
PBS	phosphate buffered saline
Pc	phthalocyanine
PDI	photodynamic inactivation
PDT	photodynamic therapy
PS	photosensitizers
PT	phototherapy
PTT	photothermal therapy
PLD	pulsed dye laser
PUT	photo-mediated ultrasound therapy
RBD	receptor binding domain
RNA	ribonucleic acid
ROS	reactive oxygen species
RRP	recurrent respiratory papillomatosis
RuV	rubella virus
RV	rotaviruses
SARS-CoV-2	severe acute respiratory syndrome coronavirus 2
SaV	sapoviruses
SWEEPS	shock wave-enhance emission photoacoustic streaming
TMPyP	tetrakis (1-methyl-4-pyridinio) porphyrin- tetra- p-toluene sulfonate
TP P2a	meso-tetraphenylporphine with two sulfonate groups on adjacent phenyl rings
UV	ultraviolet
Zn-BC-AM	Zn (II)-benzochlorine analog
